# Prevalence of celiac disease in low and high risk population in Asia–Pacific region: a systematic review and meta-analysis

**DOI:** 10.1038/s41598-021-82023-8

**Published:** 2021-01-27

**Authors:** Sara Ashtari, Hadis Najafimehr, Mohamad Amin Pourhoseingholi, Kamran Rostami, Hamid Asadzadeh-Aghdaei, Mohammad Rostami-Nejad, Mostafa Rezaei Tavirani, Meysam Olfatifar, Govind K. Makharia, Mohammad Reza Zali

**Affiliations:** 1 Gastroenterology and Liver Disease Research Center, Research Institute for Gastroenterology and Liver Diseases, Shahid Beheshti University of Medical Science, Tehran, Iran; 2 Departments of Gastroenterology, Mid Central DHB, Palmerston Hospital, Palmerston North, New Zealand; 3 Basic and Molecular Epidemiology of Gastrointestinal Disorders Research Center, Research Institute for Gastroenterology and Liver Diseases, Shahid Beheshti University of Medical Sciences, Tehran, Iran; 4 Proteomics Research Center, Faculty of Paramedical Sciences, Shahid Beheshti University of Medical Sciences, Tehran, Iran; 5 Department of Gastroenterology and Human Nutrition, All India Institute of Medical Sciences, New Delhi, India

**Keywords:** Gastroenterology, Medical research

## Abstract

This systematic review and meta-analysis study was conducted to estimate the pooled prevalence of CD in low and high risk groups in this region. Following keywords were searched in the Medline, PubMed, Scopus, Web of Science and Cochrane database according to the MeSH terms; celiac disease, prevalence, high risk population and Asian-Pacific region. Prevalence studies published from January 1991 to March 2018 were selected. Prevalence of CD with 95% confidence interval (CI) was calculated using STATA software, version 14. The pooled sero-prevalence of CD among low risk group in Asia–Pacific region was 1.2% (95% CI 0.8–1.7%) in 96,099 individuals based on positive anti-tissue transglutaminase (anti-t-TG Ab) and/or anti-endomysial antibodies (EMA). The pooled prevalence of biopsy proven CD in Asia–Pacific among high and low risk groups was 4.3% (95% CI 3.3–5.5%) and 0.61% (95% CI 0.4–0.8%) in 10,719 and 70,344 subjects, respectively. In addition, the pooled sero-prevalence and prevalence of CD in general population was significantly higher in children compared with adults and it was significantly greater in female vs. male (*P* < 0.05). Our results suggest high risk individuals of CD are key group that should be specifically targeted for prevention and control measures, and screening may prove to have an optimal cost–benefit ratio.

## Introduction

Celiac disease (CD) is a chronic autoimmune disorder which characterized by inflammation and villous atrophy (VA) in the small intestine that affects people who are genetically predisposed^1,2^. Even though the prevalence of CD varies from region to region, the average prevalence of the disease has been reported between 0.5 and 1% worldwide^3,4^. Evidence suggests that CD is higher in patients with genetic and autoimmune diseases than in healthy individuals. Prevalence of CD is high in patients with insulin dependent diabetes mellitus type 1 (DM1), chronic diarrhea, autoimmune thyroid disease (ATD), autoimmune hepatitis, Down syndrome (DS), inflammatory bowel disease (IBD), irritable bowel syndrome (IBS), Turner syndrome (TS), and first-degree relatives (FDR) of patients with CD^5,6^.

Numerous studies have been conducted in various parts of the world^7,8^, on the prevalence of CD in the general population, including in the Asia–Pacific region^4,9^. A meta-analysis of prevalence of CD in general population amongst Asian has been conducted earlier by Singh et al. however there is no reported the prevalence of CD in high risk individuals in this region. Therefore we conducted this systematic review and meta-analysis to determine and compare the prevalence of CD in high risk (first-degree relatives of patients with CD, patients with DS, DM1, ATD, IBD, dyspeptic and children and adults with symptoms frequently associated with CD such as; diarrhea and abdominal pain), and low risk (blood donors, schoolchildren and subjects without any diseases) population in Asia–Pacific region. Subgroup and meta-regression analysis were also used to address the heterogeneity between the studies in this meta-analysis.

## Results

**Figure 1:**
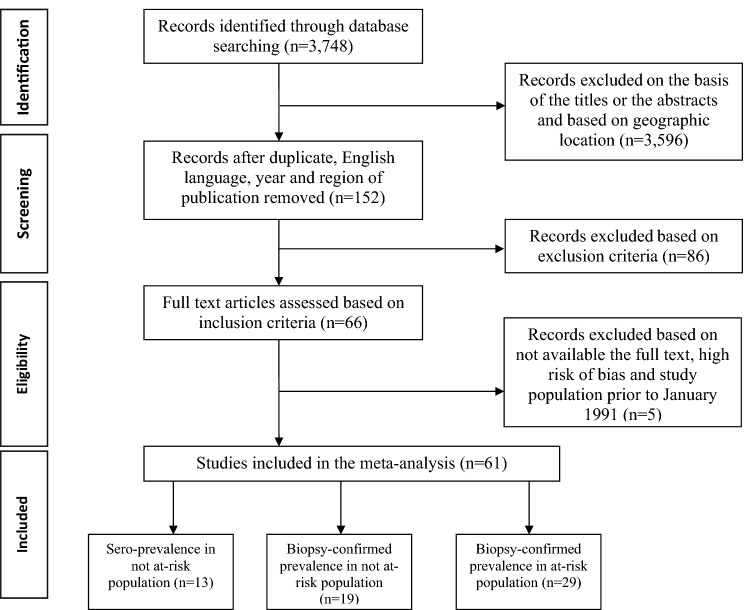
PRISMA flowchart of selecting the studies.

Our search revealed a total of 3748 articles of CD prevalence in the database. Of them, 3596 articles were rejected on the basis of the titles or the abstracts and based on geographic location. Finally, full texts of 152 articles were assessed. Eighty-six additional studies were excluded based on the inclusion and exclusion criteria. Five more studies were excluded from the study because of; unavailable full texts, high-risk of bias and study conducted prior to January 1991. Ultimately, 61 studies were included in the present meta-analysis (Fig. 1). These 61 studies reporting the sero-prevalence and prevalence of CD among low and high risk population have originated from 13 Asian-Pacific countries; New Zealand, Australia, Turkey, India, Iran, Israel, Saudi-Arabia, Arab Emirates, Kuwait, Oman, Malaysia, China and Japan. Therefore, based on our findings, we divided these countries into four geographical categories; Oceania (New Zealand and Australia), Middle-East countries (Iran, Israel, Saudi-Arabia, Arab Emirates, Kuwait, Oman and Turkey), South-Asia (India and Malaysia) and East-Asia (China and Japan).

### Pooled sero-prevalence of CD in low risk population

**Figure 2:**
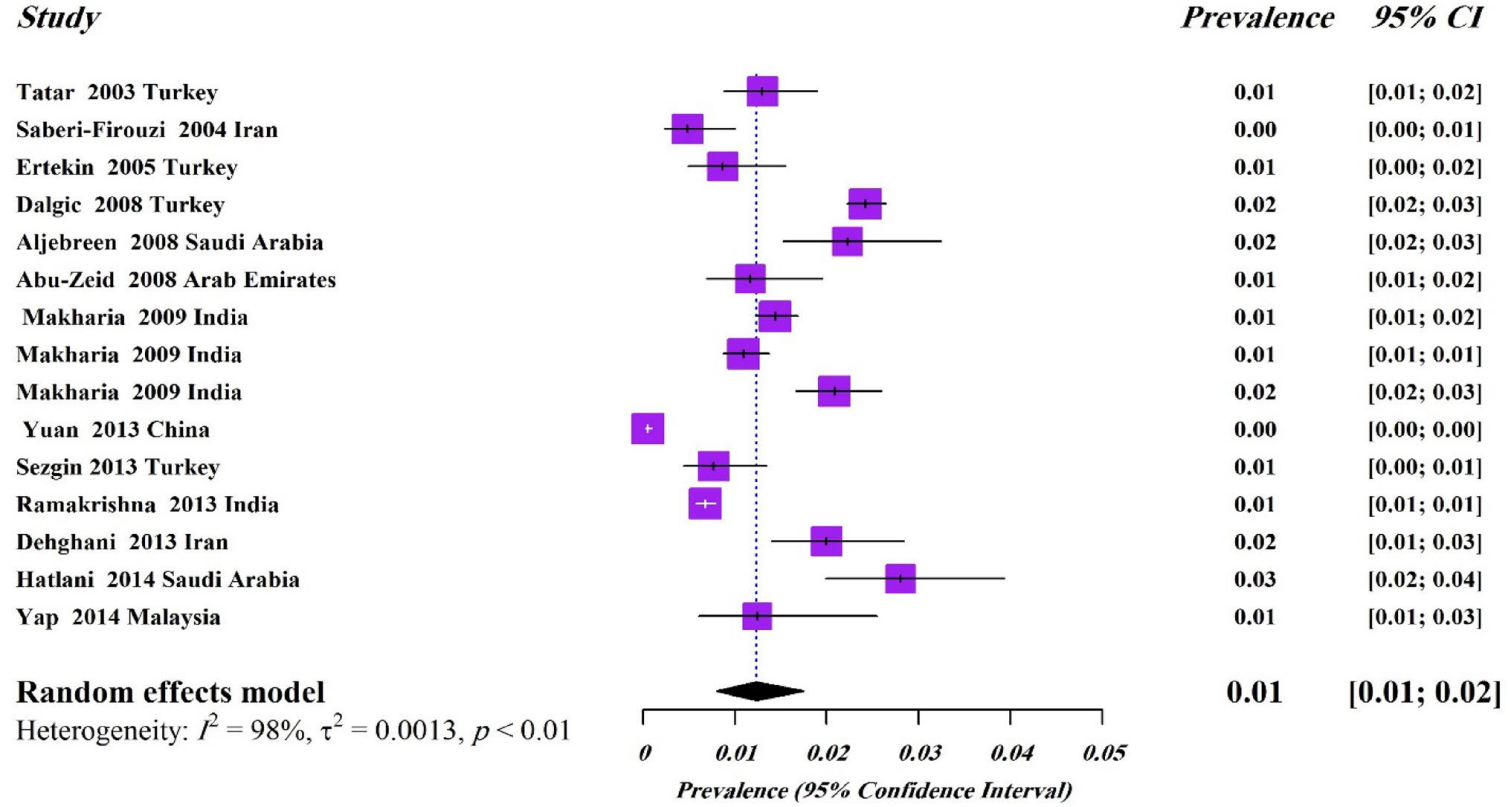
Forest plot for pooled sero-prevalence of CD in Asia–Pacific region among general population.

According to predefined criteria, 13 studies^10–22^ qualified for inclusion for estimation of the pooled sero-prevalence of CD (a positive EMA and/or antit-tTG Ab) in low risk population (Table 1). Of 96,099 included in 13 studies, 1,125 were reported to be seropositive for CD, suggesting a pooled sero-prevalence of CD to be 1.2% (95% CI 0.8–1.7%, I^2^ = 98%, *P* < 0.01) (Fig. 2). The test of heterogeneity indicated a significant heterogeneity among the studies.

**Table 1:** Sero-prevalence of CD in Asian-Pacific region among not at-risk population.

First author	Country	Region	Year of study	Population	Sample size	Age (mean)	Serology tests	Risk of bias	Sero-prevalence of CD (%)		
									Male	Female	Total
Tatar^10^	Turkey	Middle East	2001–2003	Adults	2000	33	t-TG^a,b^	Moderate	22/1914 (1.1)	4/86 (0.2)	26/2000 (1.3)
Saberi-Firouzi^11^	Iran	Middle East	2004	Adults	1440	45.5	t-TG^a^ EMA^a^	Low	–	–	7/1,440 (0.48)
Ertekin^12^	Turkey	Middle East	2005	Children	1263	11.9	t-TG^a^	Moderate	6/687 (0.87)	5/576 (0.86)	11/1,263 (0.87)
Dalgic^13^	Turkey	Middle East	2006–2008	Children	20,190	11.6	t-TG^a^ EMA^a,b^	Moderate	213/10,368 (2.05)	276/9,822 (2.81)	489/20,190 (2.42)
Aljebreen^14^	Saudi Arabia	Middle East	2007–2008	Children	1167	16.6	EMA^a^	Low	9/614 (1.46)	17/553 (3.07)	26/1,167 (2.2)
Abu-Zeid^15^	Arab Emirates	Middle East	2007–2008	Adults	1197	24.8	t-TG^a^ EMA^a^	Moderate	1/624 (0.16)	13/573 (2.27)	14/1,197 (1.17)
Makharia^16^	India	South Asia	2008–2009	*both	10,488	22.45	t-TG^a^	Moderate	68/5305 (1.28)	83/5183 (1.60)	151/10,488 (1.44)
Makharia^16^	India	South Asia	2008–2009	Adults	6845	34.4	t-TG^a^	Moderate	–	–	75/6,845 (1.10)
Makharia^16^	India	South Asia	2008–2009	Children	3643	10.5	t-TG^a^	Moderate	–	–	76/3,643 (2.06)
Yuan^17^	China	East Asia	2010–2013	Young Adults	19,778	18.8	t-TG^a^ DGP^b^	Moderate	2/13,322 (0.01)	9/6,456 (0.14)	11/19,778 (0.06)
Sezgin^18^	Turkey	Middle East	2011–2013	Adults	1554	42.1	t-TG^a,b^ DGP^a,b^	Moderate	2/772 (0.12)	10/782 (0.64)	12/1554 (0.77)
Ramakrishna^19^	India	South Asia	2011–2013	Adults	23,331	35	t-TG^a^	Low	58/10,776 (0.5)	100/12,555 (0.8)	158/23,331 (0.68)
Dehghani^20^	Iran	Middle East	2013	Children	1500	9.5	t-TG^a^	Low	–	–	30/1500 (2)
Hatlani^21^	Saudi Arabia	Middle East	2012–2014	Children	1141	11	t-TG^a^	Moderate	12/454 (2.6)	20/687 (3)	32/1,141 (2.8)
Yap^22^	Malaysia	South Asia	2012–2014	Adults	562	24	AGA^a,b^ t-TG^a,b^ EMA^a,b^	Moderate	1/238 (0.4)	6/324 (1.9)	7/562 (1.25)

*EMA* Anti-endomysial antibodies, *AGA* Anti-gliadin antibodies, *t-TG* tissue transglutaminase, *DGP* deamidated gliadin peptides.

^a^IgA.

^b^IgG.

*Adults and children together.

#### Age and gender-based difference in the sro-prevalence of CD among low risk group

Sero-prevalence of CD according to the gender was reported in 11 studies^10,12–19,21,22^. Of 45,074 males and 37,597 females, 394 and 543 subjects had CD, respectively. Pooled sero-prevalence of CD in males and females were 0.8% (95% CI 0.34–1.4%, I^2^ = 97.9%, *P* = 0.002) and 1.6% (95% CI 0.93–2.5%, I^2^ = 97%, *P* = 0.002), respectively. The pooled sero-prevalence of CD was significantly higher in females than males (*P* = 0.04). In addition, the pooled sero-prevalence of CD was significantly higher in children as compared with adults (2.04% vs. 0.95%, *P* < 0.001).

#### Geographical difference in the sero-prevalence of CD in low risk group

Of 13 studies that reported the sero-prevalence of CD in Asia–Pacific region, 9 studies were from Middle East, 3 studies from South Asia and one study was from East Asia. The highest pooled sero-prevalence in Asia–Pacific region in the Middle-East 1.4% (95% CI 0.9–2.1%), and then in South-Asia 1.2% (95% CI 0.6–2.5%) and the prevalence was least in East-Asia 0.06% (95% CI 0.03–0.09%).

### Pooled prevalence of biopsy-confirmed CD among low risk population

**Figure 3:**
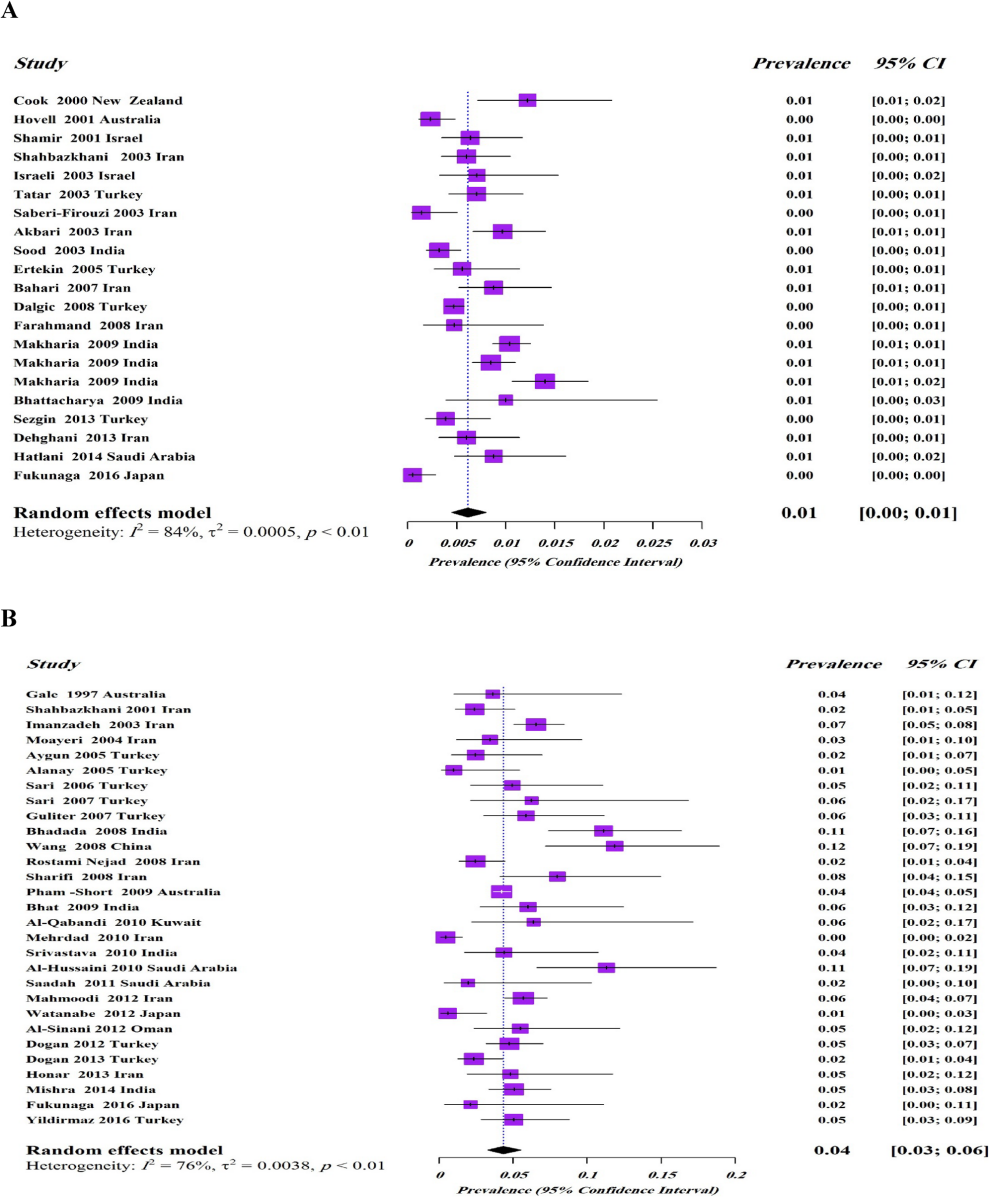
Forest plot for pooled prevalence of CD in Asia-Pacifioc region among (**A**) not at-risk population, (**B**) at-risk population.

We found 19 studies^10–13,16,18,20,21,23–33^ that reported the prevalence of biopsy-proven of CD among low risk population in Asia–Pacific region (Table 2). Of 70,344 subjects, included in 19 studies, 472 were detected to have biopsy-proven CD suggestion a pooled prevalence of biopsy-proven CD among not at-risk population to be 0.61% (95% CI 0.4–0.8%, I^2^ = 84%, *P* < 0.01) (Fig. 3A). The I^2^ test indicated significant heterogeneity among the studies.

**Table 2:** Prevalence of biopsy-confirmed CD in Asia–Pacific region among not at-risk population.

First author	Country	Region	Year of study	Population	Sample size	Age (mean)	Serology tests	Risk of bias	Prevalence of biopsy-confirmed (%)		
									Male	Female	Total
Cook^23^	New Zealand	Pacific	2000	Adults	1064	50.2	EMA^a,b^	Moderate	5/448 (1.11)	8/619 (1.29)	13/1,064 (1.22)
Hovell^24^	Australia	Pacific	2001	Adults	3011	–	EMA^a^	Moderate	–	–	7/3,011 (0.23)
Shamir^25^	Israel	Middle East	2000–2001	Adults	1571	40.7	t-TG^a^ EMA^a^	Moderate	8/1217 (0.65)	2/354 (0.56)	10/1571 (0.63)
Shahbazkhani^26^	Iran	Middle East	2003	Adults	2000	35.5	AGA^a^ EMA^a^	Moderate	10/1580 (0.63)	2/420 (0.47)	12/2000 (0.60)
Israeli^27^	Israel	Middle East	2003	Adults	850	18	t-TG^a^ EMA^a,b^	Moderate	–	–	6/850 (0.70)
Tatar^10^	Turkey	Middle East	2001–2003	Adults	2000	33	t-TG^a,b^	Moderate	12/1914 (0.62)	2/86 (2.32)	14/2000 (0.70)
Saberi-Firouzi^11^	Iran	Middle East	2004	Adults	1440	45.5	t-TG^a^ EMA^a^	Low	–	–	2/1440 (0.14)
Akbari^28^	Iran	Middle East	2003–2004	Adults	2795	33.7	t-TG^a^ EMA^a^	Low	14/1398 (1)	13/1401(0.92)	27/2,799 (0.96)
Sood^29^	India	South Asia	2003–2004	Children	4347	10.7	t-TG^a^	Low	4/2380 (0.16)	10/1,976 (0.5)	14/4,347 (0.32)
Ertekin^12^	Turkey	Middle East	2005	Children	1263	11.9	t-TG^a^	Moderate	4/687 (0.58)	3/576 (0.52)	7/1,263 (0.55)
Bahari^30^	Iran	Middle East	2006–2007	Adults	1600	33.2	t-TG^a,b^	Moderate	14/1418 (0.98)	0/182	14/1600 (0.87)
Dalgic^13^	Turkey	Middle East	2006–2008	Children	20,190	11.6	t-TG^a^ EMA^a,b^	Moderate	34/10,368 (0.33)	61/9,822 (0.62)	95/20,190 (0.47)
Farahmand^31^	Iran	Middle East	2006–2008	Children	634	12.8	t-TG^a^	Moderate	–	–	3/634 (0.47)
Makharia^16^	India	South Asia	2008–2009	Both	10,488	22.45	t-TG^a^	Moderate	48/5,305 (0.91)	61/5,183 (1.20)	109/10,488 (1.04)
Makharia^16^	India	South Asia	2008–2009	Adults	6845	34.4	t-TG^a^	Moderate	–	–	58/6,845 (0.85)
Makharia^16^	India	South Asia	2008–2009	Children	3643	10.5	t-TG^a^	Moderate	–	–	51/3,643 (1.41)
Bhattacharya^32^	India	South Asia	2009	Children	400	5.6	t-TG^a,b^	Low	1/228 (0.4)	3/172 (1.7)	4/400 (1)
Sezgin^18^	Turkey	Middle East	2011–2013	Adults	1554	42.1	t-TG^a,b^ DGP^a,b^	Moderate	0/772	6/782 (0.76)	6/1,554 (0.39)
Dehghani^20^	Iran	Middle East	2013	Children	1500	9.5	t-TG^a^	Low	4/825 (0.48)	5/675 (0.74)	9/1500 (0.6)
Hatlani^21^	Saudi Arabia	Middle East	2012–2014	Children	1141	11	t-TG^a^	Moderate	4/454 (0.9)	6/687 (0.9)	10/1,141 (0.9)
Fukunaga^33^	Japan	East Asia	2014–2016	Adults	2008	53	t-TG^a^ EMA^a^	Moderate	1/1351 (0.07)	0/657	1/2008 (0.05)

#### Age and gender-based difference in the prevalence of biopsy-confirmed CD among not low risk group

In low risk population, eight studies reported prevalence of biopsy-confirmed CD in children, 12 studies in adults, and 1 study reported the prevalence in both adults and children combined. The pooled prevalence of CD in low risk children was 0.66% (95% CI 0.4–0.9%, I^2^ = 82.3%, *P* < 0.001), in adults was 0.55% (95% CI 0.3–0.8%, I^2^ = 82.5%, *P* < 0.001) and combined adults and children was 1.04% (95% CI 0.8–1.2%). The pooled prevalence of biopsy-confirmed CD among low risk population in children was significantly higher than adults (0.6% vs. 0.5%, *P* = 0.022). The I^2^ tests indicated similar heterogeneity among the studies reporting CD prevalence for adults and children. Subgroups analyses for pooled prevalence of CD are presented in Table 4. Gender based prevalence of biopsy-confirmed CD in not at-risk group was reported in 15 studies. Pooled prevalence of biopsy-confirmed CD in not at-risk group in males and females were 0.53% (95% CI 0.3–0.7%, I^2^ = 75.5%, *P* < 0.001) and 0.74% (95% CI 0.5–0.9%, I^2^ = 48.3%, *P* < 0.001), respectively, and it was significantly higher in females than that in males (*P* = 0.04). The test of heterogeneity showed significant heterogeneity in the prevalence of CD in males and not in females (Table 4).

#### Geographical difference in the prevalence of CD in low risk group

Of the 19 studies among low risk population in Asia–Pacific region, 13 studies were from Middle-East, 3 from South-Asia, 2 from Oceania, and 1 study from East-Asia. Pooled prevalence of CD in low risk group in Oceania was 0.61% (95% CI 0.001–20%, I^2^ = 91.9%, *P* = 0.001), in the Middle-East was 0.59% (95% CI 0.4–0.7%, I^2^ = 48.8%, *P* < 0.001), in the South-Asia was 0.87% (95% CI 0.4–1.5%, I^2^ = 88.8% *P* < 0.001) and in the East-Asia was 0.05% (95% CI 0.00–0.2%). Pooled prevalence among these regions, had statistically significant difference (P < 0.001) (Table 4).

### Pooled prevalence of biopsy-confirmed CD in high risk population

Based on the inclusion criteria, we found twenty-nine studies that reported CD prevalence in high risk population in the Asia–Pacific region^33–61^ (Table 3). These studies included 10,719 subjects and of them 482 subjects were recognized with CD. Therefore, the pooled prevalence of biopsy-proven CD among at-risk population was 4.3% (95% CI 3.3–5.5%, I^2^ = 76%, *P* < 0.01) (Fig. 3B). The I^2^ test indicated significant heterogeneity among the studies.

**Table 3:** Prevalence of biopsy-confirmed of CD in Asia–Pacific region among at-risk population.

First author	Country	Region	Year of study	Population	Risk factor	Sample size	Age (Mean)	Serology tests	Risk of Bias	Prevalence of biopsy-confirmed (%)		
										Male	Female	Total
Gale^34^	Australia	Pacific	1997	Adults	DS	55	37	AGA^a^ EMA^a^	Moderate	–	–	2/55 (3.6)
Pham-Short^35^	Australia	Pacific	1990–2009	Children	DM1	4,379	6.6	t-TG^a^ EMA^a^	Moderate	147/2,147 (6.8)	38/2,232 (1.7)	185/4,379 (4.2)
Imanzadeh^36^	Iran	Middle East	1997–2003	Children	Diarrhea	825	8.5	AGA^a^ EMA^a^	Moderate	24/430 (5.6)	30/395 (7.6)	54/825 (8.9)
Al-Qabandi^37^	Kuwait	Middle East	1998–2010	Children	DM1	47	66 M	EMA, AGA^a,b^	Moderate	1/16 (6.2)	2/31(6.4)	3/47 (6.4)
Shahbazkhani^38^	Iran	Middle East	2000–2001	*Both	DM1	250	18.7	EMA^a^	Low	0/102	6/148 (4.05)	6/250 (2.4)
Moayeri^39^	Iran	Middle East	2003–2004	Children	DM1	87	11.7	t-TG^a^ EMA^a^	Moderate	1/43 (2.3)	2/44 (4.5)	3/87 (3.4)
Bhadada^40^	India	South Asia	2002–2008	*Both	DM1	189	10.8	t-TG^a^	Moderate	9/93 (9.7)	12/96 (12.5)	21/189 (11.1)
Aygun^41^	Turkey	Middle East	2005	Adults	DM1	122	–	EMA^a^	Moderate	1/54 (1.8)	2/68 (2.9)	3/122 (2.4)
Alanay^42^	Turkey	Middle East	2005	Children	DS	100	6.01	EMA^a^	Moderate	-	-	1/100 (1)
Sari^43^	Turkey	Middle East	2005–2006	Children	ATD	101	12.28	t-TG^a^	Moderate	0/11	5/90 (5.5)	5/101 (4.9)
Wang^44^	China	East Asia	2005–2008	Children	Diarrhea	118	–	EMA^a^ t-TG^a^	Low	12/85 (14.1)	2/33 (6.1)	14/118 (11.8)
Sari^45^	Turkey	Middle East	2006–2007	*Both	DM1	48	12.09	t-TG^a,b^	Low	1/18 (5.6)	2/30 (6.7)	3/48 (6.2)
Guliter^46^	Turkey	Middle East	2006–2007	Adults	ATD	136	43.1	t-TG^a^	Moderate	1/18 (5.6)	7/118 (6)	8/136 (5.9)
Rostami Nejad^47^	Iran	Middle East	2007–2008	Adults	Dyspeptic	407	36.1	t-TG^a,b^	Low	3/193 (1.5)	7/214 (3.3)	10/ 407 (2.4)
Bhat^48^	India	South Asia	2007–2009	Children	DS	100	2–18	EMA^a^ t-TG^a^	Moderate	-	-	6/100 (6)
Saadah^49^	Saudi Arabia	Middle East	2007–2011	Children	DS	51	3.58	t-TG^a^	Moderate	-	-	1/51 (2)
Sharifi^50^	Iran	Middle East	2008	*Both	DM1	100	21.8	t-TG^a^	Moderate	3/42 (7.1)	5/58 (8.6)	8/100 (8)
Mehrdad^51^	Iran	Middle East	2008–2010	*Both	ATD	454	39.4	t-TG^a^ EMA^a^	Low	0/49	2/405 (0.5)	2/454 (0.4)
Srivastava^52^	India	Middle East	2008–2010	Children	FDR	91	9.5	t-TG^a^	Moderate	–	–	4/91 (4.4)
Al-Hussaini^53^	Saudi Arabia	Middle East	2008–2010	Children	DM1	106	8.5	t-TG^a^ EMA^a^	Moderate	1/44 (2.3)	11/62 (17.7)	12/106 (11.3)
Mahmoodi^54^	Iran	Middle East	2009–2012	*Both	IBD	1,000	29	t-TG^a^	Moderate	21/497 (4.2)	36/503 (7.1)	57/1,000 (5.7)
Watanabe^55^	Japan	East Asia	2009–2012	Adults	IBD	172	43.1	t-TG^a^ DGP^a^	Moderate	1/102 (1)	0/70	1/172 (0.6)
Mishra^56^	India	South Asia	2009–2014	Adults	FDR	434	29.8	t-TG^a^	Moderate	–	–	22/434 (5.1)
Al-Sinani^57^	Oman	Middle East	2011–2012	Children	DM1	91	10.8	t-TG^a^	Moderate	2/53 (3.8)	3/38 (7.9)	5/91 (5.5)
Dogan^58^	Turkey	Middle East	2012	Adults	FDR	484	–	t-TG^a^	Moderate	–	–	23/484 (4.8)
Dogan^59^	Turkey	Middle East	2012–2013	Adults	DM1	425	37.6	EMA^a^	Moderate	7/231 (3)	3/194 (1.5)	10/425 (2.3)
Honar^60^	Iran	Middle East	2013	Children	DM1	83	10.38	t-TG^a,b^	Moderate	1/34 (3)	3/49 (6**.**1)	4/83 (4.8)
Fukunaga^33^	Japan	East Asia	2014–2016	Adults	Abdominal	47	53	t-TG^a^ EMA^a^	Moderate	1/21 (4.7)	0/26	1/47 (2.13)
Yildirmaz^61^	Turkey	Middle East	2016	Children	DM1	218	12.9	t-TG^a^	Low	6/101	5/117 (4.3)	11/218 (5)

*EMA* Anti-endomysial antibodies, *AGA* Anti-gliadin antibodies, *t-TG* tissue transglutaminase, *DGP* deamidated gliadin peptides.

*Adults and children together, *DM1* Diabetes Mellitus type1, *DS* Down syndrome, *IBD* Inflammatory bowel disease, *ATD* autoimmune thyroiditis diseases, *FDR* first-degree relatives.

^a^IgA.

^b^IgG.

#### Prevalence of biopsy-confirmed CD amongst specific diseases

Of the 29 studies among high risk population in Asia–Pacific region, DS had 4 studies, DM1 (13 studies), diarrhea (2 studies), ATD (3 studies), dyspeptic (one study), FDR (3 studies), IBD (2 studies) and abdominal pain (one study). The pooled prevalence of CD in patients with DS was 2.9% (95% CI 0.39–7.6%, I^2^ = 35.2%, *P* = 0.002), with DM1 was 5% (95% CI 3.4–6.9%, I^2^ = 64.6%, *P* = 0.002), with diarrhea was 8.4% (95% CI 0.00–58.2%, I^2^ = 71.9%, *P* = 0.002), with ATD was 2.9% (95% CI 0.00–16.7%, I^2^ = 88.9%, *P* = 0.008), with dyspeptic was 2.4% (95% CI 1.1–4.1%), with FDR was 4.8% (95% CI 4.2–5.5%, I^2^ = 1%, *P* < 0.001), with IBD was 2.6% (95% CI 0.00–87%, I^2^ = 93.7%, *P* = 0.012) and with abdominal pain was 2.1% (95% CI 0.00–8.1%). The I^2^ test indicated no significant heterogeneity among the studies in patients with DS and FDR (Table 4).

**Table 4:** Subgroup analysis for pooled prevalence of CD in Asian-Pacific region among at-risk and not at-risk populations.

	Sero-prevalence in not at-risk population				Biopsy-confirmed prevalence in not at-risk population				Biopsy-confirmed prevalence in at-risk population			
	N. patients	N. subjects	Pooled prevalence	P-value	N. patients	N. subjects	Pooled prevalence	*P*-value	N. patients	N. subjects	Pooled prevalence	*P*-value
**Population**												
Adult	310	56,707	1.01%	0.01	170	26,738	0.55%	0.02	80	2282	3.13%	0.08
Children	664	28,904	2.01%		193	33,118	0.66%		308	6396	5.28%	
Adults and children	151	10,488	1.44%		109	10,488	1.04%		97	1454	4.71%	
**Sex**												
Male	394	45,074	0.8%	0.04	163	30,345	0.53%	0.04	244	4384	4.02%	0.43
Female	543	37,597	1.65%		182	23,592	0.74%		183	5021	4.82%	
**Region**												
Oceania	–	–	–	< 0.001	20	4075	0.61%	< 0.001	187	4434	4.22%	< 0.001
Middle East	647	31,452	1.47%		215	38,538	0.59%		233	5226	4.07%	
South Asia	467	44,869	1.25%		236	25,723	0.87%		49	723	7.10%	
East Asia	11	19,778	0.06%		1	2008	0.05%		16	337	3.65%	
**Risk of CD**												
DS	–	–	–	–	–	–	–		10	306	2.90%	0.05
DM1	–	–	–	–	–	–	–		274	6145	5.06%	
Chronic diarrhea	–	–	–	–	–	–	–		68	943	8.43%	
ATD	–	–	–	–	–	–	–		15	691	2.94%	
Dyspeptic	–	–	–	–	–	–	–		10	407	2.46%	
FDR	–	–	–	–	–	–	–		49	1009	4.85%	
IBD	–	–	–	–	–	–	–		58	1172	2.62%	
Abdominal pain	–	–	–	–	–	–	–		1	47	2.13%	

*DM1* Diabetes Mellitus type1, *DS* Down syndrome, *IBD* Inflammatory bowel disease, *ATD* autoimmune thyroiditis diseases, *FDR* first-degree relatives.

#### Age and gender based difference in the prevalence of biopsy-confirmed CD among high risk group

Of these 29 studies nine studies have focused the prevalence of CD in adults, 14 studies in children and 6 studies included both adults and children. The pooled prevalence of CD in adults at high risk was 3.1% (95% CI 1.8–4.6%, I^2^ = 57.9% *P* = 0.001), in children was 5.2% (95% CI 3.7–7%, I^2^ = 57.6%, *P* = 0.002) and combined both adults and children was 4.7% (95% CI 1.2–10.1%, I^2^ = 91.6%, *P* = 0.008), respectively. There was no significant difference in the prevalence of CD amongst high risk children and adults (3.1% vs. 5.2%, *P* = 0.08). The I^2^ test indicated significant heterogeneity among the studies in three subgroups (Table 4).

The pooled prevalence of CD amongst high risk males and females in 22 studies in Asia–Pacific region was 4.02% (95% CI 2.7–5.5%, I^2^ = 68.4%, *P* = 0.003) and 4.8% (95% CI 3.3–6.5%, I^2^ = 82.1%, *P* = 0.004), respectively and there was and there was significant difference amongst males and females (*P* = 0.43). Heterogeneity tests indicated heterogeneity in the prevalence of CD for males was less than females (Table 4).

#### Geographical difference in the prevalence of CD in at risk group

Of all studies included in the present meta-analysis that reported the CD prevalence among at- risk population, 2 studies originated from Oceania, 21 from Middle-East, 3 from South-Asia, and 3 from East-Asia. The pooled prevalence of CD were 4.2% (95% CI 3.3–5.1%, I^2^ = 1%, *P* < 0.001) in Oceania, 4% (95% CI 2.9–5.3%, I^2^ = 75.2%, *P* = 0.002) in Middle-East, 7.1% (95% CI 1.3–16.7%, I^2^ = 70.4%, *P* = 0.002) in South-Asia and 3.6% (95% CI 0.00–27.7%, I^2^ = 90.7%, *P* = 0.017) in East-Asia (Table 4). The pooled prevalence of CD was significantly higher in high risk people in South-Asia as compared with the other region (*P* = 0.01). There were no other significant differences in the prevalence of CD in other regions. The I^2^ test indicated significant heterogeneity among the studies in Middle-East, South-Asia and East-Asia.

### Exploration of heterogeneity

We performed meta-regression analysis to find the source of heterogeneity among the studies in low and high risk population (Table 5). The year of the study, sample size of each study, age and gender of the subjects were used for exploration the heterogeneity. While there was an inverse association between age of the participant and the prevalence of CD in low risk group of patients (*P* = 0.02), no such association was observed in high risk population. In addition, we performed meta-analysis according to risk of bias for studies including low and moderate risk. Pooled prevalence of CD according to risk of bias and also heterogeneity test are shown in Table 6.

**Table 5:** Meta-regression analysis for exploring heterogeneity among studies.

	Not at-risk population			At-risk population		
	Coefficient	SE	P-value	Coefficient	SE	P-value
Year of study	0.0016	0.003	0.556	0.00	0.0001	0.656
Sample size	0.00	0.00	0.497	0.00	0.00	0.856
Age	− 0.0020	0.0007	0.016*	0.00	0.00	0.169
Gender	0.004	0.002	0.042*	0.00	0.00	0.433

**Table 6:** Pooled prevalence of CD according to risk of bias.

	Not at-risk population			At-risk population		
	N. study	Pooled prevalence (95% CI)	I^2^	N. study	Pooled prevalence (95% CI)	I^2^
**Risk of bias**						
Moderate	15	0.01% (0.00,0.01)	90.52%	23	0.05% (0.04,0.06)	76.84%
Low	5	0.01% (0.00,0.01)	79.03%	6	0.03% (0.01,0.06)	84.26%

### Publication bias

**Figure 4:**
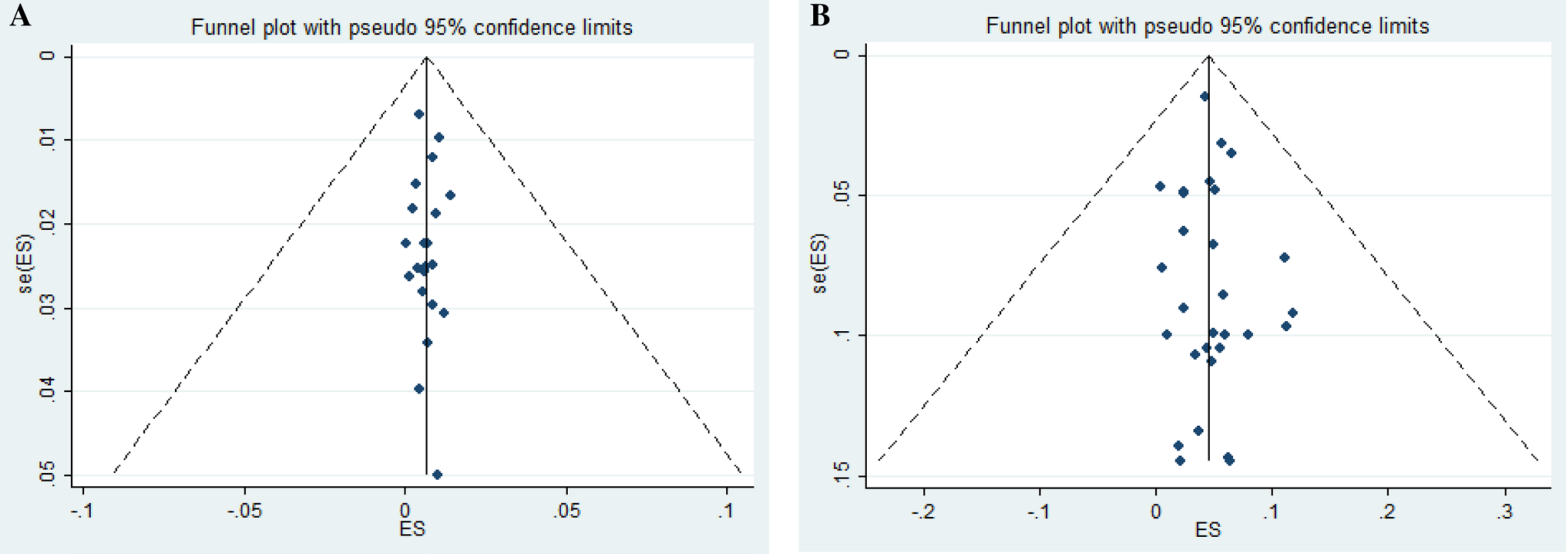
Funnel plot in not at-risk group (**A**) and at-risk group (**B**).

The result of Egger test showed presence of publication bias for studies conducted on healthy population (*P* = 0.009) and also showed presence of publication bias for studies conducted on at-risk population (*P* = 0.003). Funnel plots have shown asymmetric mood and confirmed presence of publication bias (Fig. 4).

## Discussion

To our knowledge, this is the first meta-analysis to examine the prevalence of CD in the Asia–Pacific region and to compare it between low and high risk groups. Considering that two previous studies in this region have shown only the prevalence of CD in the general population^4,9^, our findings represent the best approximation of the pooled prevalence of CD in low and high risk groups according to age (adult and children), gender (male and female) and geographical categories (Oceania, Middle-East, East-Asia, and South-Asia) in the Asia–Pacific region.

Our results revealed that the pooled sero-prevalence of CD among general population was (1.2%), and the pooled prevalence of biopsy-confirmed CD in high risk and low risk groups was (4.3%) and (0.61%) respectively. So, the pooled prevalence of CD was significantly higher in high risk population compared to low risk subjects (*P* < 0.001). Sero-prevalence and biopsy-confirmed prevalence in Asian-Pacific countries varied from 0.06% in Turkey to (2.8%) in Saudi Arabia and (0.05%) in Japan and 1.4% in India, respectively. The analysis of CD prevalence within 4 geographical categories of Oceania, Middle-East, East-Asia, and South-Asia showed the highest prevalence of CD among low and high risk population was in the South-Asia (0.8%) and (7.1%), respectively. While, the highest sero-prevalence of CD was reported in Middle-East countries (1.4%).

Our findings suggest that CD is a much greater problem in the Asia–Pacific region than has previously been appreciated. The prevalence of CD in this region, both in low and high risk groups, was similar and comparable to its prevalence in Europe and the United States^62–64^. Our results showed that the pooled prevalence of CD among FDR (4.8%) in 1009 individual, were similar to those reported in previous studies in the US and Europe between (4.5%) and (10%)^64–68^. We found that the prevalence of CD in ATD patients was higher compared with the general population (2.9% vs. 0.6%) and that the risk of CD can be increased by about 4–5 times in ATD subjects. This is slightly higher than global pooled prevalence of biopsy proven CD (1.6%) reported by Roy et al.^69^ in 6024 subjects with ATD^69^. The pooled prevalence of CD among patients with DM1 in this study was (5%) that extracted from 13 studies on 6,145 patients with DM1. A study carried out in Sweden reveled the globally pooled prevalence of CD in DM1 patients was (6%) based on 27 studies on 26,605 DM1 patients^70^. In addition, they reported the pooled prevalence of CD among DM1 patients; (6.1%) in Europe, (4.8%) in North America and (4.8%) in four countries in Middle-East and Oceania (Saudi Arabia, Iran, India and Australia)^70^. Our results were slightly lower than in European countries but similar to North America. Furthermore, the pooled prevalence of CD among subjects with DS (2.9%) in this study was lower than pooled CD prevalence in DS patients that reported by Du et al. (5.8%) based on 31 studies from Europe (21 studies), United States (6 studies) and Asia–Pacific region (4 studies) in 4383 individuals^71^. The pooled prevalence of CD among DS patients in the study by Du et al. was (6%) in Europe, (5.7%) in America and (4.5%) in Asia–Pacific countries (India, Australia, Saudi Arabia and Israel)^71^.

In addition, we evaluated the prevalence of CD in children and adults with symptoms associated with CD incudes, diarrhea and abdominal pain. The pooled prevalence of CD in patients with chronic diarrhea estimated 8.4% in the study based on two studies from Iran and China on 942 children^36,44^. According to the data presented in the study, CD is common among patients labeled as chronic diarrhea especially in children. Given that CD may be missed or diagnosed late in children with chronic diarrhea, immunological screening with the subsequent morphologic study of the small intestine is recommended to all patients with the chronic diarrhea syndrome to enable the early diagnostics of CD^72^.

Our analyses revealed a significant heterogeneity in prevalence of CD among low and high risk groups from different countries in Asia–Pacific region. To explore this heterogeneity we examined subgroups of studies such as year of study, sample size, age and gender. Meta-regression analysis has confirmed that CD prevalence in low risk groups decreased with age at testing and female gender. While, in high risk population did not found any association between age or gender and prevalence of CD in Asia–Pacific region. The prevalence of CD in high risk adults was significantly higher than in children, suggesting a link between the duration of gluten consumption and the development of an immune response to gluten. Therefore, heterogeneity was substantially reduced when sero-prevalence and prevalence of CD in not at-risk populations was calculated separately for men/women and adult/children. The heterogeneity reported in the prevalence of CD in this study is partly due to methodological differences between studies which include the type of diagnostic (serology/biopsy test) and study population (adults/children). It is likely that prevalence of CD also varies from country to country in Asia–Pacific, because of diverse dietary practices and prevalence of predisposing HLA-DQ2/HLA-DQ8 haplotypes in the general population^9,73^.

While the present study reports a pooled prevalence of CD in Asian-Pacific region among low and high risk population for the first time, this meta-analysis has a few limitations too. Studies on the prevalence of CD in general population are available only from 13 countries in this region. Therefore, the lack of population-based prevalence data from many countries (Azerbaijan, Kazakhstan, Turkmenistan, Kyrgyzstan, Tajikistan, Cambodia, Vietnam, Mongolia, Hong Kong, Sri Lanka, Myanmar, Maldives, Nepal, and Bhutan) in the Asia–Pacific region is a major limitation. Another limitation was the most studies in this region reported the prevalence of CD based on the serology and even if the biopsies were performed in seropositive individuals, only small proportion of patients underwent biopsies. So, we had to exclude a lot of studies based on our inclusion and exclusion criteria.

In conclusion, we have undertaken the first meta-analysis study in low and high risk population in the Asia–Pacific region. Our results suggest that CD is common in Asian-Pacific region and pooled sero-prevalence and prevalence of biopsy-confirmed CD in low risk groups was 1.2% and 0.6%, respectively, which is similar to Western countries. In addition, the prevalence of CD in high risk population was significantly higher than low risk group (4.3% vs. 0.6% P < 0.001). High risk individuals of CD are key group that should be specifically targeted for prevention and control measures, and screening may prove to have an optimal cost–benefit ratio.

## Methods

We developed a protocol, including eligibility criteria, search strategies, criteria for study selection and methods for extracting data according to the Preferred Reporting Items for Systematic Review and Meta-Analysis (PRISMA) guidelines^74^.

### Search strategy

Previously published papers indexed in Medline (National Library of Medicine), PubMed, Scopus, Web of Science (Thomson Reuters; New York, USA), and Cochrane Library (Cochrane Collaboration; Oxford, United Kingdom) were searched for this systematic review and meta-analysis with the following MeSH terms and keywords: “Celiac diseases”, “Coeliac disease” and “Prevalence” alone or combination. To find prevalence of CD among high risk population search strategy was based on the words of CD prevalence in patients with “diabetes mellitus type 1”, “chronic diarrhea”, “autoimmune thyroid disease”, “Down syndrome”, “inflammatory bowel disease”, “dyspepsia”, and “first-degree relatives with CD”. Each one was cross-referenced with “Asia–Pacific region” and countries in this region such as Australia, New Zealand, India, Pakistan, Turkey, Iran, etc. The first recommendations for diagnosis of CD were published by the European Society for Pediatric Gastroenterology Hepatology and Nutrition (ESPGHAN) in 1990^75^, which we considered this year as a dividing year for well-defined diagnostic criteria for CD and other gluten-related disorders. All related articles published between January 1991 and March 2018 were, therefore, included in this review. The search for the studies was performed in English and this analysis did not include those without access to the full text. Moreover, in order to conclude the qualifying studies, all reference lists of relevant publications were also reviewed and the retrieved references were also disregarded due to duplication. To exclude unrelated studies with no eligibility requirements, the names, abstracts, as well as full texts were carefully read.

### Countries covered

Asian-Pacific is a region of the world in or near the Western Pacific Ocean. The region varies in area depending on which context, but it typically includes much of East Asia, South Asia, Southeast Asia, Central Asia, Oceania and Pacific. To cover the entire Asia in this study, West Asia was also examined in this paper. Therefore, based on our purpose, Asian-Pacific region was divided into 5 sub-regions; East Asia (China, Mongolia, North Korea, South Korea, and Japan), South-Central Asia (Tajikistan, Uzbekistan, Kazakhstan, Turkmenistan, Kyrgyzstan, Sri Lanka, Bangladesh, India, Afghanistan, Pakistan, Islamic Republic of Iran, Bhutan, Nepal, and the Maldives), Southeast Asia (Brunei, Cambodia, Indonesia, Laos, Malaysia, Myanmar, Philippines, Singapore, Thailand, and Vietnam), West Asia (Georgia, Armenia, Azerbaijan, Turkey, Cyprus, Syria, Lebanon, Israel, Palestine, Jordan, Iraq, Kuwait, Bahrain, Qatar, Saudi Arabia, United Arab Emirates, Oman, and Yemen), and Oceania (Australia and New Zealand).

### Diagnostic criteria for CD

The diagnosis of CD was based on a combination of at least one positive celiac-specific serological tests such as anti-tissue transglutaminase (anti-t-TG) antibodies, anti-endomysial antibodies (EMA) and deamidated gliadin peptides (DGP) antibodies, anti-gliadin antibody (AGA) and all confirmation villous atrophy by duodenum biopsy according to Marsh classification^76^. In addition, studies that reporting the sero-prevalence of CD in the healthy population (having a positive t-TG, EMA and DPG antibodies without biopsy confirmation) were analyzed separately.

### Inclusion criteria

For evaluating the prevalence of CD; all population based studies reporting the prevalence of CD in not at-risk population and hospital registries studies for at-risk population in Asia–Pacific region were recorded.

### Exclusion criteria

The exclusion criteria were as following: (a) studies documenting the prevalence based on self-reporting (b) studies that reporting the CD prevalence by only (AGA) marker (c) Case Report, Case Series and Letter to Editor Studies were excluded (d) studies without access to the full text and those with unclear results were excluded.

### Study selection and quality assessment

Two authors (A.S and NM.H) performed the literature search, reviewed all the full texts, and individually evaluated the articles based on pre-decided inclusion and exclusion criteria. Moreover, the risk of bias was calculated using the risk of bias tool for prevalence studies developed by Hoy et al.^77^. Based on this tool, studies were assessed for external and internal validity using a 10-point checklist and grouped into a low, moderate, or high risk of bias. The studies with a score of less than 6 were considered to have a high risk, 6 to 8 was considered a moderate risk, and 9 to 10 was considered a low risk of bias. The studies with a high risk of bias were excluded from the present meta-analysis. Disagreements between two authors were resolved by discussion. In case disagreements persisted, third author (RN.M.) reviewed the study and made the final decision. To increase the quality of the review, a blind method was used hiding the authors name and name of the journal.

### Data extraction

Information was extracted separately about the sero-prevalence and biopsy confirmed prevalence of CD in at-risk and not at-risk populations in adults and children. Information contained the name of the first author, year of publication, place of study, demographic characteristics of study participants including; number, sex and age, the type of serological tests and duodenal biopsy. Based on our inclusion criteria, finally 61 articles including 19 articles on CD prevalence in not at-risk population, 29 on CD prevalence among at-risk population and 13 articles on sero-prevalence of CD in not at-risk population in English language from January 1991 to March 2018, which reported the prevalence or sero-prevalence of CD in Asia–Pacific region, were entered in this study.

### Pooled prevalence and sero-prevalence of CD

Only studies in which 50 percent or more of seropositive individuals (those with positive anti-tTG and/or AEA) underwent a biopsy were included to measure the pooled prevalence of CD. The 50 percent discontinuity value was chosen because we assumed that the real prevalence of biopsy-proven CD was wrongly reduced among the studies in which less than 50 percent of positive individuals were subjected to biopsy. For the estimation of pooled sero-prevalence only, studies in which less than 50% of seropositive individuals underwent a biopsy were included.

### Statistical analysis

We obtained pooled prevalence and sero-prevalence of CD in not at-risk and at-risk population, separately. Pooled prevalence of CD was obtained based on the proportion of individuals with CD and its confidence interval in each study. Prevalence was calculated assuming binomial distribution. In addition, we calculated prevalence of CD for subgroups such as region or sex. CD prevalence between groups was compared using chi-square test. For all pooled prevalence, the random model was used. I^2^ statistics was employed to evaluate heterogeneity among studies. I^2^ value > 50% was denoted as high heterogeneity. We applied the fixed effect model when the data were homogeneous. When the cause of heterogeneity was not known, the random effect model was used. To explore the sources of heterogeneity, meta-regression analysis was done. Moreover, Begg’s test was carried out for recognizing publication bias. All analyzes performed by STATA 14.0 (STATA Corp; College Station, Texas, USA) software and significant level was considered as 0.05.
